# Fracture Analysis of Highly Flexible Adhesives: Cohesive Zone Modelling across a Wide Spectrum of Temperatures and Strain Rates

**DOI:** 10.3390/polym16162383

**Published:** 2024-08-22

**Authors:** Tomas Nunes, Maria J. P. Ribas, Alireza Akhavan-Safar, Ricardo J. C. Carbas, Eduardo A. S. Marques, Sabine Wenig, Lucas F. M. da Silva

**Affiliations:** 1 Faculdade de Engenharia, Universidade do Porto, Rua Dr. Roberto Frias, 4200-465 Porto, Portugal; up202105919@edu.fe.up.pt (T.N.); up201806409@edu.fe.up.pt (M.J.P.R.);; 2 Institute of Science and Innovation in Mechanical and Industrial Engineering (INEGI), Rua Dr. Roberto Frias, 4200-465 Porto, Portugal; 3 Sika Automotive AG, Kreuzlingerstrasse 35, 8590 Romanshorn, Switzerland

**Keywords:** highly flexible adhesives, cohesive zone modelling, strain rate, temperature, mode I fracture

## Abstract

This study focuses on the prediction of the fracture mechanics behaviour of a highly flexible adhesive (with a tensile elongation of 90%), since this type of adhesive is becoming widely used in automotive structures due to their high elongation at break and damping capacity. Despite their extensive applications, the understanding of their fracture mechanics behaviour under varying loading rates and temperatures remains limited in the literature. In addition, current prediction models are also unable to accurately predict their behaviour due to the complex failure mechanism that such bonded joints have. This study aims to determine whether a simple triangular cohesive zone model (CZM), which predefines the crack path, can reproduce the load–displacement curves of adhesives under various temperatures and strain rates. To achieve this, a calibrated CZM is used, adapting the model for reference joints and then validating it with independent test results conducted in a wide range of loading and environmental conditions. The tests were performed at speeds between 0.2 and 6000 mm/min and at three different temperatures ranging from −30 °C to 60 °C. Mode I fracture toughness was measured using the DCB (double cantilever beam) specimens. Using a simple triangular CZM may not be optimal for predicting the mechanical response of highly flexible adhesives with complex failure mechanisms and multiple crack paths. However, by correctly adjusting the cohesive zone properties for a limited set of reference conditions, it is possible to accurately predict the mechanical response of these joints across various test speeds and temperatures, significantly reducing costs and effort.

## 1. Introduction

The use of adhesive bonding is becoming increasingly prevalent in numerous industrial sectors, including aerospace, automotive, and construction. This is due to the numerous advantages it offers over traditional joining methods such as welding and riveting [1,2]. Adhesive bonding has the potential to offer several benefits, including the ability to join materials that would otherwise be incompatible, the potential for weight reduction, improved stress distribution, and the elimination of stress concentrations associated with mechanical fasteners [3,4]. Furthermore, adhesive bonding can result in a more aesthetically pleasing finish and enable the creation of complex, integrated structures [5,6].

Polyurethane adhesives have gained considerable popularity within the automotive industry, largely due to their exceptional properties and adhesion capabilities. This preference can be attributed to the excellent characteristics exhibited by these adhesives, particularly at higher strain rates. Polyurethane adhesives are employed in a wide range of applications, including the bonding of dissimilar materials for structural purposes. Such materials are frequently subjected to environmental conditions that can lead to deterioration and bond attenuation, including factors such as dampness, vibrations, and temperature differences, among others [7,8].

One of the critical factors influencing the performance of adhesive joints is temperature [9]. The mechanical properties of adhesives can undergo significant alterations in response to changes in temperature, which can have a profound impact on the overall strength and durability of the joint [10]. It is of paramount importance to comprehend the influence of temperature on the functionality of adhesive joints, as this knowledge is instrumental in guaranteeing the dependability and security of adhesive-bonded structures, particularly in instances where the joints may be subjected to a vast array of environmental conditions [11,12].

In addition to temperature, several other factors can influence the mechanical properties of adhesive joints, including challenging environments [13,14], moisture [15], the presence of additives, and joint geometry [5,6]. While extensive research has been conducted on the effects of these parameters on various adhesive families, there is a relative lack of studies focusing specifically on polyurethane adhesives [3,11]. This represents a significant research gap, given that polyurethane adhesives are gaining more prominence in a variety of industries due to their excellent flexibility/ductility, and resistance to environmental factors [4,5].

Regarding tensile and shear properties, Borges et al. [16] concluded that both tensile and shear strength increase with increasing loading rate. However, Jia et al. [17] found a similar type of behaviour at room temperature, but this was not so linear for other temperatures. In fact, the stiffness of the adhesive even decreased while increasing loading rates at negative temperatures. In the study conducted by Machado et al. [18], it was observed that an increase in temperature led to a decrease in tensile strength, while an increase in strain rate led to an increase in tensile strength. Some authors sought to ascertain whether there existed any methodology that might influence the results of the tests conducted in the study of fracture behaviour. In a separate study, Nunes et al. [19] examined a novel test procedure where DCB tests were conducted with the crack tip at a constant strain rate throughout the test. This approach was found to be more consistent than tests performed at a constant test speed, as evidenced by a reduction in the standard deviation of the test results. The same author evaluated the strain rates experienced in an adhesive layer during testing with two regularly used fracture toughness specimen designs, DCB and ENF. The major goal was to understand how adhesive characteristics affect the evolution of strain rate. The results showed that the strain rate at the bond line varied significantly during DCB testing with a consistent crosshead displacement rate. For example, the strain rate increased twofold at the start of the test when compared to the end [20]. Despite the small number of studies undertaken, fracture behaviour remains a vastly unknown field due to the inconsistent results produced. Viana et al. [21] examined the strain rate dependency of a crash-resistant epoxy at low and high temperatures. Their findings showed that the energy absorbed by the specimens (SLJ) in quasi-static circumstances decreased with temperature, which was due to the drop in adhesive yield stress with temperature. Jia et al. [22,23] reported a similar phenomenon, in which the critical fracture energy reduced as the loading rate increased. However, in mode I, the crucial energy release rate decreased with a temperature drop for the quasi-static state, but *G_IIc_* dropped with a temperature increase for higher strain rates. This feature was not examined in the study undertaken by Borges et al. [24]. The goal of this study was to create a finite element model (the cohesive zone model, which was also evaluated by Tserpesa et al. [25]) that accurately depicts the mechanical behaviour of adhesives under mode I fracture conditions, while also considering the influence of characteristics on strain rate. As strain rates rose, the two adhesives tested showed an increase in ultimate stress and critical energy release rate. Bidadi et al. [26] intended to investigate the effect of loading rate in greater detail and confirm its influence on the mixed-mode fracture behaviour of an epoxy resin material. The results show that raising the loading rate greatly reduces fracture loads and related fracture resistance values for each mode mixity. This was owing to a reduction in the size of the crack tip fracture process zone as loading rates increased. Borges et al. [16] did not, however, confirm that the energy release rate for all mode mixities increased with loading rate for both adhesives examined. Perez et al. [27] sought to fill a research gap in the strain rate and temperature impacts of polyurethane adhesives, with findings indicating that raising the loading rate greatly enhanced the maximum strength of DCB specimens, with the greatest sensitivity reported at ambient temperature.

Despite the wealth of research on the effects of various parameters on the mechanical properties of adhesive joints, there remains a significant research gap regarding the modelling and prediction of the behaviour of polyurethane adhesives. Although polyurethane adhesives are employed in a multitude of industrial applications, the existing literature on the influence of various parameters, including temperature, loading rate, and moisture, on their mechanical properties is relatively limited. This lack of understanding impedes the capacity to accurately predict the performance of polyurethane adhesive joints under varying conditions, which is of paramount importance for ensuring the reliability and safety of adhesive-bonded structures.

The primary objective of this study is to develop a methodology for calculating the load–displacement curves of a highly flexible adhesive within a joint under varying conditions, including temperature and loading rate, using cohesive zone modelling (CZM). Although a triangular CZM is generally applied to less ductile materials, this study posits that a precisely calibrated triangle-shaped CZM, despite not being directly related to the underlying physics, can effectively reproduce the behaviour of highly flexible adhesives with complex failure mechanisms and multiple crack paths. To achieve this, a comprehensive matrix of experimental conditions was developed, with a subset of these conditions used to calibrate the numerical model under extreme conditions. It was assumed that an averaging technique could predict conditions between the calibration conditions. Once the numerical model has been calibrated, the cohesive properties for the remaining conditions are determined by averaging the values of neighbouring conditions. This provides a robust and versatile approach for predicting the mechanical behaviour of highly flexible adhesive joints under a wide range of operating conditions.

## 2. Experimental Details

This section outlines the manufacturing process and testing procedures employed to conduct the DCB tests. Any subsequent references to the QS, ISR and HSR nomenclature are relative to the quasi-static condition (0.2 mm/min), intermediate strain rate (200 mm/min) and high strain rate (6000 mm/min), respectively. For the purposes of simplicity when referring to test temperatures, the terms LT, RT and HT will be utilised to signify low temperature (−30 °C), room temperature (23 °C) and high temperature (60 °C), respectively. The selected conditions were chosen based on the prospective applications of the adhesive in question. The experimental details are explained in the following sections.

### 2.1. Materials and Manufacturing Process

In order to conduct the experimental procedure, DCB specimens were manufactured using high-strength steel (PM300) substrates. The mechanical properties of a two-component highly flexible adhesive, modified for industrial use, were investigated. The adhesive has a chemical base consisting of a polyol as the soft segment and an isocyanate as the hard segment, with a mixing ratio by volume of 100:100. Table 1 presents the physical and mechanical properties of the adhesive.

The tensile properties were determined by evaluating bulk specimens shaped in accordance with the dogbone configuration. The adhesive plate was manufactured by pouring an uncured material into a rectangular mould and pressing it between steel plates. A silicone rubber frame was utilised to contour the material, thereby controlling its thickness. Moreover, the frame functioned to contain the adhesive within the mould, thereby providing hydrostatic pressure. In order to facilitate the removal of the adhesive plate, two silicone sheets were placed above and below the silicone frame. This prevented the adhesive from adhering to the metal plates of the mould. Subsequently, the mould was closed and placed within a hydraulic press at 30 bars, with the objective of reducing the formation of air bubbles (voids) that could potentially arise from the mixing of the two-component adhesive. Once the adhesive plate had fully cured, it was machined into a dogbone shape in accordance with the specifications set forth in British Standard BS 2782.

**Figure 1:**
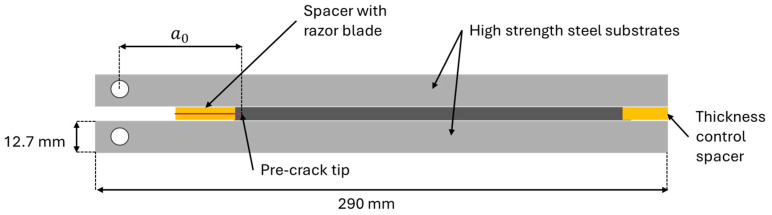
DCB joint scheme.

In accordance with the standard ASTM D-3433-99, the DCB specimens were manufactured with the requisite geometry, Figure 1, for all conditions evaluated under fracture mode I loading [28]. Following the specifications provided by the adhesive’s manufacturer, an optimal thickness was identified to ensure the best mechanical performance. Consequently, the joints were tested at this optimal thickness. Surface preparation was crucial to enhance adhesion between the substrate and the adhesive. Prior to the application of the adhesive, the substrate surfaces were sandblasted to remove any traces of iron oxides, resulting in a surface more conducive to adhesion and reducing the risk of interfacial failure. The surface was then degreased with acetone and primed to enhance adhesion quality [29].

After a 24-hour period, the specimens coated with primer were positioned in a standard mould used for the fabrication of DCB joints. The specimens were held in place by top and bottom plates with holes for guide pins, enabling the adhesive to be applied. Due to the viscosity and high thickness of the bondline, 3D-printed parts were required on the sides to ensure an adequate quantity of adhesive remained in the joint during the curing process. A razor blade with a thickness of 0.1 mm was employed to create an initial crack length of 45 mm in the centre of the adhesive layer. The crack size was measured from the loading point. The blade was sandwiched between two spacers with controlled thicknesses to guarantee the adhesive’s thickness during the manufacturing process and to ensure that the blade was positioned at the centre of the adhesive layer. Additionally, a spacer with the same thickness as the adhesive layer was positioned at the end of the joint, as illustrated in Figure 1.

Following the manufacturing process, the curing cycle commenced at room temperature and lasted seven days, with no further need for post-curing.

### 2.2. Testing Plan

All experiments were conducted using an Instron 8801 servo-hydraulic testing machine (Instron, Norwood, MA, USA), equipped with a load cell rated for ±100 kN. For testing under extreme temperatures, a thermal chamber was employed, with temperature control facilitated by a thermocouple affixed directly to the adhesive layer. The standard deviations for all conditions assessed in this study were calculated based on the repeatability of three samples per condition.

Bulk samples were subjected to tensile loading until failure. Given the impracticality of using an extensometer for strain measurement at extreme temperatures, digital image correlation (DIC) was employed as an alternative. This technique involved capturing the test with a high-resolution camera, followed by frame-by-frame analysis using GOM software to accurately measure strain.

To calibrate the model, four reference conditions were chosen. These were low and high temperatures, both the quasi-static and the high strain rate. The results of the tensile tests carried out on these can be found in Table 2.

## 3. Data Reduction Approach

Data reduction methods traditionally depend on monitoring the crack length as it propagates, as noted in the literature. However, this approach can lead to significant errors in calculating fracture energies, making it less viable. Consequently, the Compliance-Based Beam Method (CBBM) was employed in this study as a more suitable alternative. For the adhesives tested, a fracture process zone (FPZ) forms ahead of the main crack tip due to the nucleation of multiple microcracks across the adhesive thickness, coupled with the damage and plasticity occurring within this zone. An extensive FPZ can obscure the precise location of the crack tip, complicating the determination of the actual crack position. If data reduction methods do not account for FPZ effects, the estimated crack size during testing will be inaccurate, placing the estimated crack tip well ahead of the true crack tip [30]. In highly ductile materials, a substantial amount of energy is dissipated within the FPZ, which impacts the measured toughness [30]. Traditional methods fail to account for this energy dissipation, whereas CBBM does. Derived from linear elastic fracture mechanics, CBBM does not require direct measurement of crack length during testing, as it infers the equivalent crack length solely through the evaluation of specimen compliance. Using this method, the mode I fracture energy is given by

(1) $$G_{I}=\frac{6P^{2}}{B^{2}h}\left(\frac{{{2a}_{eq}}^{2}}{h^{2}E_{f}}+\frac{1}{5G}\right)$$

where *P* is the load, *B* is the specimen width, *h* is the thickness of the substrates, *G* is the shear modulus of the substrates, $a_{eq}$ is the equivalent crack length and $E_{f}$ is the corrected flexural modulus required as this method does not account for stress concentrations and substrate rotation near the crack tip [30].

## 4. Numerical Method

### 4.1. Cohesive Zone Modelling (CZM)

The concept of cohesive behaviour was first introduced by Barenblatt [31] and Dugdale [32] to describe the gradual occurrence of local fracture processes around crack tips as a progressive phenomenon occurring within the cohesive zone. The implementation of CZM enables the replication of crack initiation and propagation, thus facilitating the detailed analysis of crack initiation, growth, and interaction. This provides a macroscopic reproduction of damage along a predefined crack path. The CZM approach simulates the evolution of strength and subsequent softening, leading up to complete failure, thus allowing for the gradual degradation of material properties to be incorporated into the simulation.

CZMs are related to traction laws that describe the evolution of strength and softening of the material. In general, triangular-shaped curves are easier to model and more suitable for brittle materials. For ductile adhesives, trapezoidal shapes are particularly effective, as they accurately capture the plastic flow of the adhesive at the transition from elastic to plastic behaviour [33]. However, trapezoidal CZMs required at least two more parameters to defined compared to the triangle CZM. On the other hand, using this approach is not as straightforward as the triangle one, as a trapezoidal CZM is not an in-built model in FE programmes like Abaqus.

**Figure 2:**
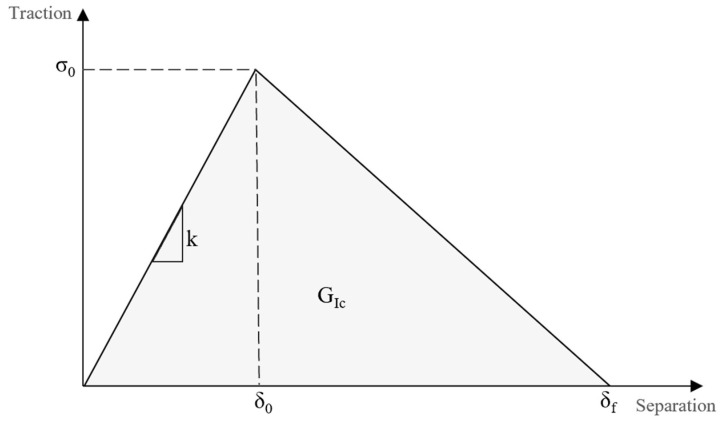
Triangular traction–separation law.

A typical triangular traction separation law relationship, as shown in Figure 2, can be divided into two regions. The first is linear elastic, which persists until the peak of cohesive strength (*σ*_0_) is reached at *δ*_0_. The second is a region of softening, where damage initiation will commence in conjunction with the reduction in the stiffness of the cohesive element. The slope of the linear elastic portion represents the normal cohesive stiffness (*k*), while the area under the curve denotes the fracture energy (*G_IC_*). Once the cohesive strength has diminished within the softening region at *δ_f_*, the crack will propagate along the failure path. This method provides critical insights into failure mechanisms during fracture tests, enhancing the design and testing of more resilient materials and structures. It facilitates the advancement of knowledge regarding the behaviour of materials under stress, thereby enabling the development of innovative solutions to prevent material failure [34,35]. In this study, a triangular shape law was employed for predicting the load–displacement curves of the joints under study.

### 4.2. Numerical Modelling

**Figure 3:**
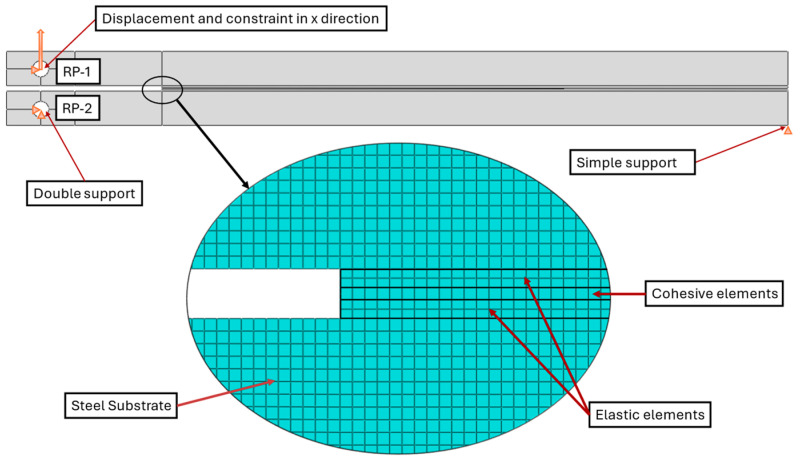
DCB model sections, boundary conditions and mesh.

The specimen’s geometry is illustrated in Figure 3. The adhesive layer was divided into elastic and cohesive elements. Due to the high thickness of the adhesive and the limitation of the application of the CZM method to high-thickness layers, it was necessary to make that division [36]. The middle layer presents cohesive elements, representing the zone where the crack will propagate within the simulation. The values of the elastic properties of the cohesive zone were divided by 0.5, which corresponds to its thickness.

A quasi-static type of analysis was defined for the step-in question. Consequently, a quasi-static test with displacement control was conducted. In terms of parameter measurement, the field output enables the distribution of measurement data throughout the entire model, including stress and damage. Localised parameters can be quantified through the utilisation of a history output. The reaction force was quantified at RP-1, along with the displacement. These values were required for the subsequent calculation of the load–displacement curves.

Three boundary conditions were derived from experimental tests. A displacement was defined for the reference point of the pin of the upper substrate (RP-1), as well as a constraint in the x-direction. Regarding the pin at the lower substrate (RP-2), a constraint was imposed in both the x- and y-directions. Furthermore, the lower substrate was also prevented from moving in the y-direction. To accurately simulate the load on the pins of the apparatus used in experimental tests, it was also necessary to define the coupling interactions between the reference points and the surface of the holes.

Regarding the mesh, quad elements were applied to each zone with an approximate size of 0.5 mm. For each section, a plane stress element type family was employed, with the exception of the cohesive zone, where a cohesive zone family was utilised with a sweep technique. In order to facilitate convergence of the problem, the viscosity of the cohesive elements was adjusted to 1E-5, while the default viscosity was retained for the substrate and adhesive solid elements [33].

### 4.3. Simulation Approach

The objective of the simulations was to develop a methodology for triangular-shaped laws with the aim of analysing load–displacement behaviour curves for flexible adhesives with multiple crack paths under varying loading conditions. To this end, a test matrix was constructed, incorporating variations in loading rate and test temperature. The calibration conditions involve the selection of the four reference conditions shown in Table 3. A series of simulations were conducted for the calibrating conditions, with varying values of *k*, *σ*, and *G_IC_* applied to the properties of the cohesive zone. Once the numerical curve had been fitted to the experimental data, the final values were established. The experimental curves used for this fitting correspond to the average load–displacement curve of the experimental ones to the considered situation. These curves are presented in the following section when analysing the experimental results. Subsequently, the cohesive zone properties for the remaining conditions were determined by averaging the values of the neighbouring conditions. The values were then applied to the model, resulting in simulations being carried out for each of the remaining validation conditions in the matrix. Load–displacement curves were subsequently obtained.

This suggested approach allows for the calibration of a model with calibrating conditions make it useful for prediction of the load–displacement curves of the joints subjected to a wide range of loading and temperature conditions.

## 5. Results and Discussion

### 5.1. Experimental Results

**Figure 4:**
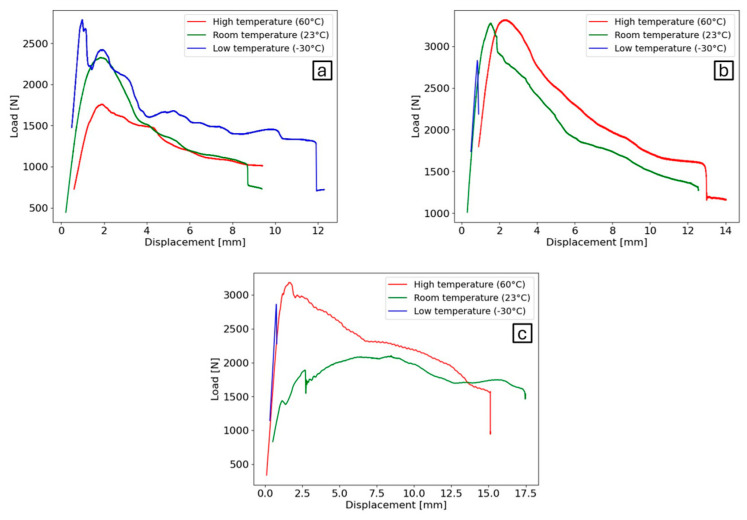
Average experimental load–displacement curves of DCB joints under different temperatures for (**a**) quasi-static loading (0.2 mm/min), (**b**) intermediate-strain-rate loading (200 mm/min), (**c**) high-strain-rate loading (6000 mm/min).

Figure 4 shows the average experimental load–displacement curves, at different temperatures and loading rates. Results from the load–displacement curves show that for every loading rate the lower the temperature, the stiffer the behaviour of the adhesive tends to be. This is since the temperature is closer to the *T_g_* of the material (−45 °C), where molecule movement slows down and there is a shift from a flexible, rubber-like state to a rigid, glassy state. At quasi-static conditions, the adhesive displays optimal behaviour at lower temperatures, with the maximum load and displacement until failure is exhibited. An increase in temperature at quasi-static conditions results in a decrease in the load capacity of the adhesive as well as the displacement to failure, with minimal variation observed between RT and HT in terms of displacement. Additionally, stiffness tends to decrease with higher temperatures, with a more pronounced decline in the adhesive stiffness at HT. The observed increase in the displacement rate of the tests has led to the conclusion that the adhesive at LT behaves in a more fragile manner. At higher strain rates, the adhesive failed completely after achieving the maximum load, exhibiting minimal ductility, and behaving more like an epoxy. While the maximum strength capacity does not appear to be significantly affected by the displacement rate increase, the maximum displacement until failure does decrease abruptly. In the case of higher temperatures, both the strength and displacement of the joints were enhanced by the increase in loading rate. The maximum displacement exhibited less sensitivity to temperature fluctuations, with results at RT and HT showing similar values for each loading rate condition. The joints tested at HT exhibited a greater increase in strength and displacement with the increase in test speed. During the testing of RT at higher speeds, a decline in performance was observed, with a notable reduction in the adhesive’s maximum load-bearing capacity. The results indicate that the adhesive displays superior performance when subjected to temperatures close to *T_g_* at quasi-static conditions. However, when subjected to accelerated testing, the optimal conditions for enhanced performance were temperatures considerably higher than the *T_g_*.

The values of critical energy release rate under mode I (*G_IC_*) for these conditions are presented in Table 4.

### 5.2. Failure Mechanism

Defects within the adhesive or at the interface between the adhesive and the adherends can serve as initiation points for cracks. These defects can include voids, inclusions, or weak interfaces. When a crack encounters such a defect, it may either propagate through the adhesive or be deflected into the adherent material.

**Figure 5:**
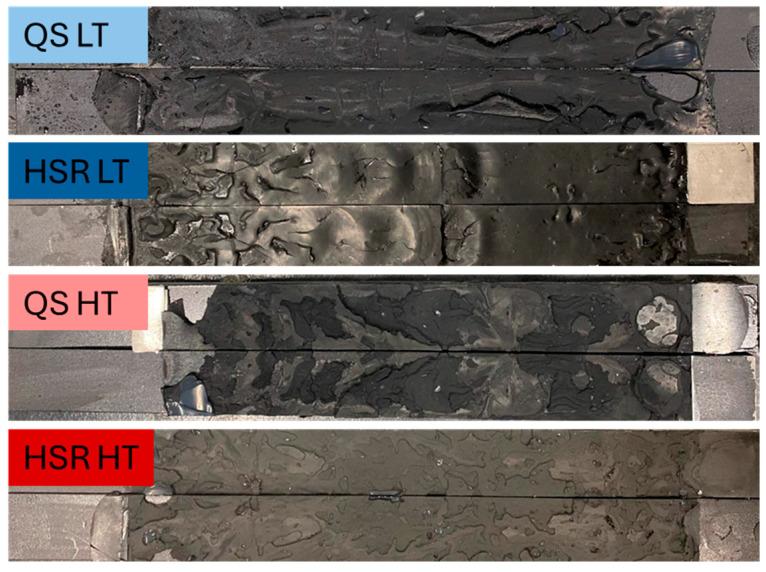
Fracture surfaces of the pivotal conditions. QS and HSR stand for quasi-static (0.2 mm/min) and high strain rate (6000/min), respectively, and LT and HT correspond to low temperature (−30 °C) and high temperature (60 °C), respectively.

Figure 5 shows some of the specimens tested under critical conditions to calibrate the model. For all DCBs, the crack appears to propagate more towards the centre of the adhesive layer, as a similar distribution of adhesive can be seen in both substrates. Under quasi-static loading conditions, the fracture surfaces at both low and high temperatures were similar. However, under high loading rate conditions, the fracture surfaces differed significantly between the low and high temperature tests. At low temperature and high strain rate, the surface roughness changed and showed some spherical clusters of the adhesive that appeared to have detached from one of the substrates close to the pre-crack zone. A much smoother and cleaner distribution of adhesive can then be seen, showing a more uniform distribution of adhesive with increasing load rate and apparently with decreasing temperature, which is to be expected as the adhesive becomes more brittle when both of these conditions are met [37].

At low temperatures, adhesives generally become more brittle. Brittle materials tend to fracture with less plastic deformation, leading to smoother fracture surfaces. The lack of significant plastic deformation prevents the formation of rough, uneven surfaces that are typically seen in ductile fractures. Also, at lower temperatures, the viscosity of the adhesive may increase, leading to a more uniform distribution as the adhesive does not flow or move as much during the bonding process. This uniformity can result in a more even fracture surface when the joint fails.

Regarding the high loading rate, this may induce a brittle fracture even in materials that might otherwise exhibit some ductility. The rapid application of load does not allow enough time for the material to undergo plastic deformation, resulting in a cleaner and smoother fracture surface.

### 5.3. Numerical Results

**Figure 6:**
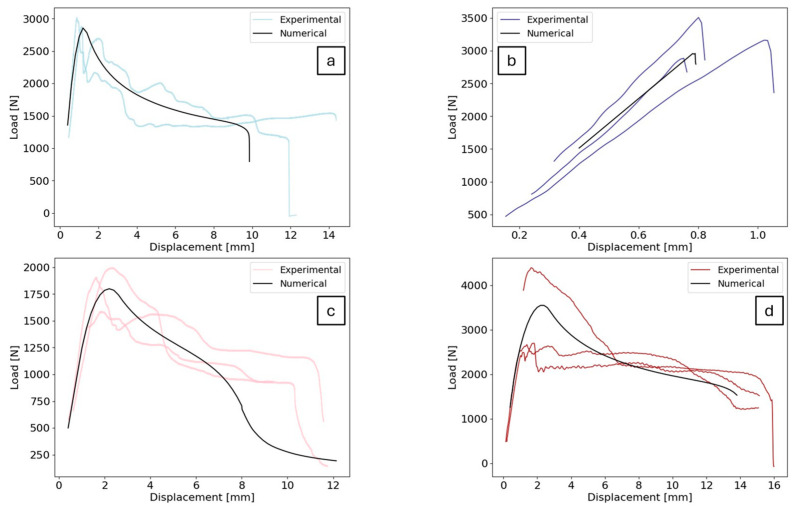
Comparison of numerical load–displacement curve and experimental deviations for (**a**) low-temperature (−30 °C) and quasi-static conditions (0.2 mm/min), (**b**) low-temperature (−30 °C) and high-strain-rate conditions (6000 mm/min), (**c**) high-temperature (60 °C) and quasi-static conditions (0.2 mm/min), (**d**) high-temperature (60 °C) and high-strain-rate conditions (6000 mm/min).

The iterative process, which employed an inverse CZM approach, permitted the adjustment of cohesive property values until the numerical load–displacement curves aligned with the experimental data. The outcomes of the calibration conditions are presented in Figure 6, while the properties that were attained to achieve these outcomes are detailed in Table 5.

The numerical simulations indicated a satisfactory fit, with the curves remaining within the experimental deviations for most of the displacement. The cohesive zone models used in simulations may not capture all aspects of the complex fracture process, potentially resulting in discrepancies between numerical and experimental maximum displacement. At quasi-static conditions, this is especially observed. It should also be noted that the cohesive zone model tends to be conservative, frequently underestimating the maximum displacement in comparison to the experimental results.

Knowing the parameters of the four previously selected conditions, the values for the cohesive properties of the other conditions were calculated. The values obtained are shown in Table 6.

**Figure 7:**
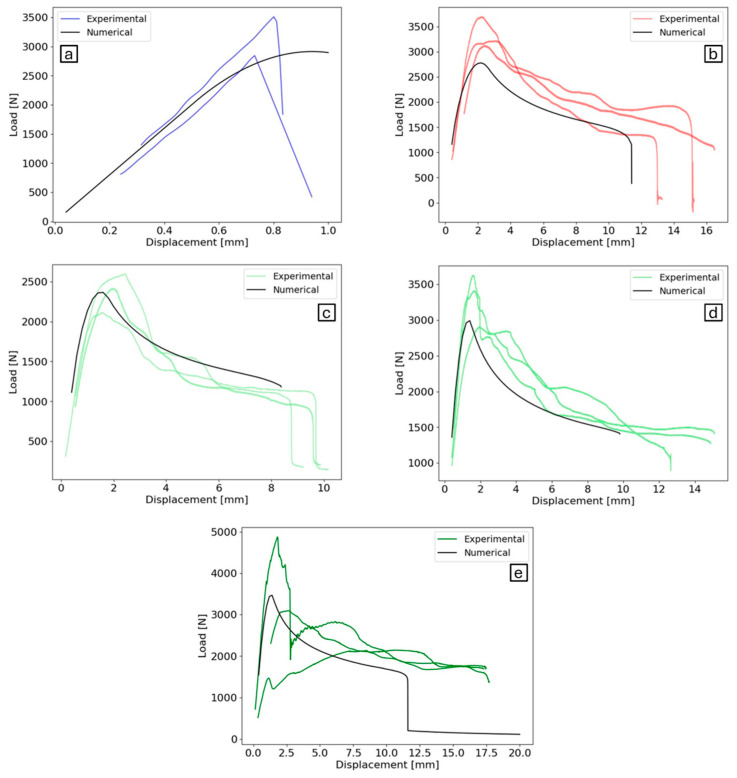
Validation of the CZM for different independent test conditions at various loading rates and temperatures. The curves show the comparison of numerical and experimental load–displacement curves for (**a**) low-temperature (−30 °C) and intermediate-strain-rate conditions (200 mm/min), (**b**) high-temperature (60 °C) and intermediate-strain-rate conditions (200 mm/min), (**c**) room-temperature (23 °C) and quasi-static conditions (0.2 mm/min), (**d**) room-temperature (23 °C) and intermediate-strain-rate conditions (200 mm/min), (**e**) room-temperature (23 °C) and high-strain-rate conditions (6000 mm/min).

The aforementioned values were employed to predict the load–displacement curve of the joints. The results are illustrated in Figure 7. It should be noted due to the brittle response of the joint at ISR-LT conditions, only the crack initiation regarding joint failure is considered as the failure criterion.

Although a triangular CZM is typically used for less ductile materials, this study demonstrated that a precisely calibrated CZM, despite not being directly related to the underlying physics, can be effectively applied to highly flexible adhesives with complex failure mechanisms and multiple crack paths. Additionally, the calibrated CZM can be used for the same joint tested across a wide range of loading rates and temperatures. According to the results, the numerical load–displacement curves tend to be slightly conservative. The peak loads from numerical simulations closely match the experimental data, as do the displacements at which these occur. Regarding the maximum displacement the adhesive can withstand, the model is slightly conservative, with experimental curves extending approximately 25% further in terms of displacement.

The CZM provided a useful representation of the load–displacement curve using a straightforward method, which is beneficial for design purposes. However, one limitation was identified regarding the failure mechanism, which differs from the observed phenomenon in practice. Consequently, it is not possible to ascertain the critical point in that regard.

## 6. Conclusions

The study of numerical methods for predicting the behaviour of materials under various loading conditions is of great importance, as it provides valuable insights into the durability and load capacity of materials. The utilisation of CZM, a highly valuable numerical tool for the investigation of crack initiation and propagation in a diverse range of materials, particularly adhesives, permitted this study to examine the efficacy of a distinct methodology employing CZM in the prediction of the shape of load–displacement curves for a highly flexible adhesive. The employment of CZMs with triangular shape laws is typically confined to brittle materials with defined crack paths. However, this study demonstrated that with an alternative methodology, it can be utilised for ductile materials with multiple crack paths. Indeed, by modifying the cohesive zone properties of the numerical model for a limited number of reference conditions, insights were gained regarding the anticipated displacement curves of the adhesive for a wide range of temperatures and loading rates.

The numerical results exhibited a satisfactory alignment with experimental data, indicating that the model employed is somewhat conservative. Peak loads, joint stiffness, and the displacement at crack propagation onset, the numerical simulations closely matched the observed values in the experimental data. This is evidenced by the maximum displacement at joint failure predicted by the model being approximately 25% less than that observed experimentally. The congruence between the numerical and experimental data substantiates the versatility of this method in accommodating diverse loading conditions within the calibration range.

Despite the lack of a direct physical link between the properties employed in this approximation and the problem under consideration, the approximation itself provides valuable insights into the load–displacement curves under various loading conditions. Furthermore, the approximation necessitates a relatively small quantity of experimental data in comparison to the insights it provides. As only a limited number of test results are required as reference conditions and only a minimal effort is needed to manipulate the properties, this approach greatly reduces the experimental and numerical effort that would be required with other approaches.

## Figures and Tables

**Table 1:** Physical and mechanical properties of the studied adhesive.

Properties	Values
$\mathrm{Glass}\;\mathrm{transition}\;\mathrm{temperature},\;T_{g}$ (°C)	−45
Maximum tensile strength (MPa)	14.5 ± 1.5
Maximum tensile strain	0.9 ± 0.1
Young’s modulus (MPa)	265.5 ± 18.5

**Table 2:** Tensile properties of the calibrating testing conditions.

**Tensile Properties**	**LT (−30 °C)**		**HT (60 °C)**	
	QS (0.2 mm/min)	HSR (6000 mm/min)	QS (0.2 mm/min)	HSR (6000 mm/min)
Maximum stress [MPa]	30.9 ± 0.2	22.1 ± 0.5	7.8 ± 0.4	9.1 ± 1.3
Young’s modulus [MPa]	1102 ± 32	7311 ± 343	105.4 ± 4.0	162.3 ± 3.0

**Table 3:** Calibration (reference) and validation conditions.

	Loading Rate	Quasi-Static (0.2 mm/min)	Intermediate Strain Rate (200 mm/min)	High Strain Rate (6000 mm/min)
Temperature				
Low temperature (−30 °C)		$G_{IC}$✓ *k*✓ *σ*✓ Calibration	Validation	$G_{IC}$✓ *k*✓ *σ*✓ Calibration
Room temperature (23 °C)		Validation	Validation	Validation
High temperature (60 °C)		$G_{IC}$✓ *k*✓ *σ*✓ Calibration	Validation	$G_{IC}$✓ *k*✓ *σ*✓ Calibration

**Table 4:** Experimental values of critical energy release rate under mode I for different temperature and strain rates.

*G_IC_* (N/mm)	Quasi-Static (0.2 mm/min)	Intermediate Strain Rate (200 mm/min)	High Strain Rate (6000 mm/min)
Low temperature (−30 °C)	4.8±0.2	3.5±0.3	3.8±0.2
Room temperature (23 °C)	4.0±0.1	6.1±0.7	9.0±0.4
High temperature (60 °C)	3.3±0.2	9.7±0.7	10.3±0.2

**Table 5:** Cohesive zone properties defined numerically for the reference conditions to obtain a good fit with the experimental curves.

Test Speed and Temperature	*G_IC_* (N/mm)	*k* (MPa)	*σ* (MPa)
0.2 mm/min at −30 °C (QS/LT)	8.0	100.0	2.4
0.2 mm/min at 60 °C (QS/HT)	2.8	8.0	1.9
6000 mm/min at −30 °C (HSR/LT)	15.0	65.0	1.6
6000 mm/min at 60 °C (HSR/HT)	8.0	324.0	5.0

**Table 6:** Predicted cohesive zone properties for the independent validation test conditions.

Test Speed and Temperature	*G_IC_* (N/mm)	*k* (MPa)	*σ* (MPa)
200 mm/min at −30 °C (ISR/LT)	11.5	82.5	2.0
200 mm/min at 60 °C (ISR/HT)	5.4	166.0	3.3
0.2 mm/min at 23 °C (QS/RT)	5.4	54.0	2.2
6000 mm/min at 23 °C (HSR/RT)	11.5	194.5	3.3
200 mm/min at 23 °C (ISR/RT)	8.5	124.3	2.7

## Data Availability

Data is included in the paper.
